# Hypertension in sub-Saharan Africa: the current profile, recent advances, gaps, and priorities

**DOI:** 10.1038/s41371-024-00913-6

**Published:** 2024-05-02

**Authors:** Lebo F. Gafane-Matemane, Ashleigh Craig, Ruan Kruger, Omotayo S. Alaofin, Lisa J. Ware, Erika S. W. Jones, Andre Pascal Kengne

**Affiliations:** 1 Hypertension in Africa Research Team, North-West University, Potchefstroom, 2520 South Africa; 2 SAMRC Research Unit for Hypertension and Cardiovascular Disease, North-West University, Potchefstroom, 2520 South Africa; 3 SAMRC/Wits Developmental Pathways for Health Research Unit (DPHRU), University of the Witwatersrand, Soweto, 1864 South Africa; 4 Division of Nephrology and Hypertension, Groote Schuur Hospital and Kidney and Hypertension Research Unit, University of Cape Town, Cape Town, South Africa; 5 Non-Communicable Diseases Research Unit, South African Medical Research Council, Francie Van Zijl Dr, Parow Valley, Cape Town, 7501 South Africa; 6 Department of Biological and Environmental Sciences, Faculty of Natural Sciences, Walter Sisulu University, Mthatha, South Africa

**Keywords:** Risk factors, Hypertension

## Abstract

Recent global and regional reports consistently confirm the high and increasing prevalence of hypertension in sub-Saharan Africa (SSA), with poor detection, treatment, and control rates. This narrative review summarises the burden of hypertension in SSA and recent findings from community-based hypertension management strategies. We further outline prominent risk factors according to recent data and associated underlying mechanisms for hypertension development. An extensive review of literature showed that most countries have reported on the prevalence of hypertension during 2017–2023, despite limitations linked to the lack of nationally representative studies, heterogeneity of sampling and data collection methods. Task-shifting approaches that assign roles to model patients and community health workers reported improved linkage to healthcare services and adherence to medication, with inconsistent findings on blood pressure (BP)-lowering effects over time. The regularly reported risk factors include unhealthy diet, sedentary lifestyle, increased adiposity and underweight, ageing, level of education, and/or income as well as psychosocial factors. Newer data on the pathophysiological mechanisms leading to hypertension and potential areas of intervention are reported from children and adults and include, among others, salt-handling and volume overload, endothelial function, BP dipping patterns and the role of human immunodeficiency virus . To conclude, significant strides have been made in data reporting from SSA on the burden of hypertension in the region as well as biomarker research to improve understanding and identification of areas of intervention. However, gaps remain on linkage between knowledge generation, translation, and implementation research. Coordinated studies addressing both discovery science and public health are crucial to curb hypertension development and improve management in SSA.

## Introduction

Elevated blood pressure (BP) remains the largest, preventable risk factor for premature death globally, with about 10.8 million deaths in 2019 attributed to raised BP [1]. Although the age-standardised prevalence of hypertension has stabilised between 1999 and 2019, its prevalence continues to soar in low and middle-income countries (LMICs) accounting for three-quarters of the global number of people living with hypertension [2, 3]. In sub-Saharan Africa (SSA), the prevalence of hypertension has increased, reaching 48% (CI. 42–54%) in women and 34% (CI. 29–39%) in men in 2019 [2]. Despite numerous calls to action and road maps by regional and international bodies, awareness, treatment and control ( < 10% in men and 13% in women) remains dismally low, with disparities between ethnicities and within countries, rural versus urban areas as well as between men and women [2–6].

The main drivers for the high prevalence of hypertension in SSA emanate from the transition from a traditional lifestyle to a more westernised lifestyle [7–10]. While the frequency of risk factors for hypertension and cardiovascular disease (CVD) has been generally lower in rural than in urban populations [7, 8], recent data from SSA show that the gap is closing as rural populations embrace modern lifestyle [11, 12]. Hypertension risk factors include dietary habits consisting of a high intake of salt, sugar, and fat and a low intake of fruits and vegetables, sedentary lifestyles, smoking and alcohol use, obesity, and sociodemographic factors [13–15]. While knowledge generation has increased on the effect of these risk factors on pathophysiological mechanisms underlying the elevation in BP over the life course, there are still significant gaps. Therefore, more research is needed into the mechanisms of hypertension development to inform preventative and therapeutic interventions.

This review aims to: (1) summarise the burden of hypertension in SSA and findings from community-based management strategies, (2) outline current knowledge of risk factors and associated underlying mechanisms and (3) identify knowledge gaps that need to be addressed to improve understanding of hypertension development and reduce the treatment and control gaps.

## Methods

In this manuscript, we adopted a narrative review approach to evaluate the current profile of hypertension in SSA including prevalence, treatment, and control rates as well as community-based management strategies. Literature search of relevant articles published between January 1, 2017, and June 30, 2023, on MEDLINE, ERIC, PubMed, EBSCOhost, Health Source - Consumer Edition, CINAHL with full text, Health Source: Nursing/Academic Edition, Google Scholar, Academic Search Complete, African Journals Online (AJOL) and online grey literature. These electronic databases were searched for peer-reviewed articles evaluating the prevalence, treatment and control of hypertension using a strategy that combined free text keywords with Medical Subject Heading (MeSH) phrases (Supplementary Table 1). Eligibility criteria included articles which have reported on hypertension prevalence, risk factors, management strategies, articles published in English language or other languages where an English translation is accessible and lastly peer-reviewed articles published between January 1, 2017, and June 30, 2023 with study settings in SSA. We excluded review articles, meta-analyses, systematic literature reviews, editorials, conference papers and articles without full text.

One of the authors conducted a comprehensive literature search with the combination of the keywords highlighted in the Supplementary Table 1. After removing duplicates and ensuring that the eligibility criteria were met, two authors independently conducted title, abstract and article screening on selected peer-review articles for appropriateness. The third author then checked and confirmed that the extracted data were accurate and resolved any discrepancies arising from article selection. In total, 41 studies were included in the review to evaluate the current profile of hypertension in SSA and four included for the community-based management strategies that used mixed methods and/or cluster randomised trial study designs. Data extraction included the study settings, name of author and publication year, study design, and sample size which are presented in Table 1. Other variables are prevalence, awareness, treatment, and control of hypertension. We present an overview of the findings from the articles included in the review using a narrative synthesis. We further extracted information on possible mechanisms of hypertension development from the articles reporting on hypertension prevalence/treatment/management to determine to what extent these articles included this aspect to inform new treatment approaches. This was in addition to recent original papers addressing mechanisms associated with hypertension development in SSA populations. In addition to the purpose of presenting recent information, the reporting period was selected to avoid the overlap with previous notable reviews of hypertension in Africa in the last decade [16–18].

**Table 1:** The prevalence, awareness, treatment and control of hypertension in sub-Saharan Africa published during 2017–2023.

Country	Author, published (yr)	Data collection (yr)	Cohort	Survey tool	Hypertension prevalence (%)			Hypertension awareness (%)			Hypertension treatment (%)			Hypertension control (%)			Study type / setting	Score	Survey tool / study limitation
					Overall	Men	Women	Overall	Men	Women	Overall	Men	Women	Overall	Men	Women			
***Southern Africa***																			
Angola	Pereira et al., 2021 [22]	2019	*n* = 7112; 18 yrs+	MMM	38.6	−	−	59.8	−	−	50.6	−	−	20.8	−	−	Cross-sectional. Screened 6/18 provinces.	C	Selection bias (self-selected participants). Insufficient funds to carry out extensive screening. Inability of follow-up (single visit). Non-representative nature of the survey.
Botswana	Tlhakanelo et al., 2021 [24]	2019	*n* = 5459; 18 yrs+	MMM	32.1	−	−	44.8	−	−	41.5	−	−	19.5	−	−	Cross-sectional. Screened 4 locations/villages.	C	Selection bias (self-selected participants). Insufficient funds to carry out extensive screening. Inability of follow-up (single visit). Non-representative nature of the survey.
Eswatini	Geldsetzer et al., 2019 [60]	2014	*n* = 3183;15–69 yrs	WHO STEPS	29.8	−	−	−	−	−	21.0	−	−	7.0	−	−	Cross-sectional. Nationally representative / non-weighted data.	A	None reported for the data presented on this specific country.
Lesotho	Egede et al., 2021 [41]	2014	*n* = 5356; 15–49 yrs	DHS	15.3	−	−	−	−	−	−	−	−	−	−	−	Cross-sectional. Survey tool was nationally representative. Sampling weights were used to account for complex survey design and allow weighting to the population.	A	Hypertension was self-reported and based on prior diagnosis. Data was collected between the years 2010 and 2016, therefore population level changes in the prevalence of hypertension may have changed.
Mozambique	Jessen et al., 2019 [46]	2017	*n* = 3846; 18 yrs+	MMM	31.1	−	−	−	−	−	73.4	−	−	38.4	−	−	Cross-sectional. Screened in the capital city (Maputo).	C	Selection bias (self-selected participants). Insufficient funds to carry out extensive screening. Inability of follow-up (single visit). Non-representative nature of the survey.
Namibia	Craig et al., 2018 [47]	2013	*n* = 3068; 35–64 yrs	Namibian DHS	44.6	44.3	44.9	46.6	39.4	51.3		33.8	42.1	−	12.6	18.9	Cross-sectional. First national-level health survey.	A	The sample of respondents from whom objective assessments were measured was limited to those 35–64 years of age, narrowing the representativeness of estimates. BP was measured on single visit.
South Africa	Kandala et al., 2021 [7]	2016	*n* = 8230; 15 yrs +	South African DHS	48.2	18.5	30.1	−	−	−	−	−	−	−	−	−	Cross-sectional. A sampling frame was used for the 2016 DHS. Data was weighted.	A	BP measurements were not taken at the same time of the day for all participants due to the large numbers of participants surveyed.
Zambia	Goma et al., 2021 [58]	2019	*n* = 9232; 18 yrs+	MMM	30.7	−	−	42.6	−	−	27.6	−	−	9.7	−	−	Cross-sectional. Screened in capital city (Lusaka).	C	Selection bias (self-selected participants). Insufficient funds to carry out extensive screening. Inability of follow-up (single visit). Non-representative nature of the survey.
Zimbabwe	Tozivepi et al., 2021 [59]	2019	*n* = 350; 18 yrs+	WHO STEPS		−	−	−	−	−	−	−	−	−	43.4	40.4	Cross-sectional. Screened hospital patients admitted at 1 hospital in 1 province.	C	The type and length of medication being used by participants were not investigated in this study.
***West Africa***																			
Benin	Houehanou et al., 2021 [23]	2019	*n* = 3637; 18 yrs+	MMM	37.5	−	−	64.5	−	−	43.9	−	−	34.9	−	−	Cross-sectional. Screened 13 sites in 6 regions.	C	Selection bias (self-selected participants). Insufficient funds to carry out extensive screening. Inability of follow-up (single visit). Non-representative nature of the survey.
Burkina Faso	Cissé et al., 2021 [21]	2013	*n* = 4628; 25–64 yrs	WHO STEPS	17.9	9.5	8.4	17.5	13.8	21.1	−	−	−	−	−	−	Cross-sectional. National screening.	A	BP was measured on single visit.
Cabo Verde	Azevedo et al., 2021 [26]	2019	*n* = 17,627; 18 yrs+	MMM	34.0	−	−	68.4	−	−	51.3	−	−	23.8	−	−	Cross-sectional. Carried out throughout the national territory.	A	Selection bias (self-selected participants). Insufficient funds to carry out extensive screening. Inability of follow-up (single visit). Non-representative nature of the survey.
Cote d’Ivoire	Euloge et al., 2019 [32]	2017	*n* = 24,563; 18 yrs+	MMM	20.4	−	−	−	−	−	4.4	−	−	44.6	−	−	Cross-sectional. Screened 55 sites (mainly in Abidjan).	C	Selection bias (self-selected participants). Insufficient funds to carry out extensive screening. Inability of follow-up (single visit). Non-representative nature of the survey.
Ghana	Twumasi-Ankrah et al., 2021 [37]	2019	*n* = 7102; 18 yrs+	MMM	25.9	−	−	36.5	−	−	30.0	−	−	13.8	−	−	Cross-sectional. Screened 75 sites across 4 regions.	C	Selection bias (self-selected participants). Insufficient funds to carry out extensive screening. Inability of follow-up (single visit). Non-representative nature of the survey.
Guinea	Balde et al., 2017 [38]	2014	*n* = 1100; 15–64 yrs	WHO STEPS	50.3	53.5	46.5		−	−	−	−	−		−	−	Cross-sectional. Screened lower Guinea (15% of the total population).	C	No limitations pertaining to BP measurement or data was noted.
Guinea-Bissau	Turé et al., 2021 [39]	2021	*n* = 995; 18–69 yrs	WHO STEPS	26.9	29.6	24.2	54.5	48.7	54.5	55.1	48.7	55.1	54.0	45.5	54.0	Cross-sectional. Screened 50 sampling units out of 408 geographical locations in the capital city. Data was weighted based on age and sex distribution of the city.	C	BP measured on single visit. Participants within each household were not randomly selected.
Gambia	Cham et al, 2018 [36]	2010	*n* = 3573; 25–64 yrs	WHO STEPS	29.0	27.7	30.5	28.0	14.0	28.6	−	−	−	−	−	−	Cross-sectional. Screened local government and enumeration areas (20 households were selected from each enumeration area).	A	BP measured on single visit. Participants with any missing BP values were excluded (biased estimates). Salt intake – known hypertension risk factor – was not included in this survey.
Liberia	−	−	−	−		−	−	−	−	−	−	−	−	−	−	−	–	E	–
Mali	Bâ et al., 2018 [44]	2012–2013	*n* = 2102; 15–65 yrs	WHO STEPS	23.5	−	−	−	−	−	−	−	−	−	−	−	Cross-sectional. Screened 30 clusters. Community-based data.	C	No limitations pertaining to BP were noted.
Niger	Toure et al., [48]	2017–2019	*n* = 2297; 18 yrs+	MMM	33.2	−	−	−	−	−	3.4	−	−	−	−	−	Cross-sectional. Screened in only 1 site (Niamey).	C	Selection bias (self-selected participants). Insufficient funds to carry out extensive screening. Inability of follow-up (single visit). Non-representative nature of the survey.
Nigeria	Wahab et al., 2021 [49]	2019	*n* = 2065; 18 yrs+	MMM	39.2	−	−	62.9	−	−	55.4	−	−	25.9	−	−	Cross-sectional. Screened 6 geopolitical zones.	C	Selection bias (self-selected participants). Insufficient funds to carry out extensive screening. Inability of follow-up (single visit). Non-representative nature of the survey.
Senegal	Benzekri et al.,2018 [51]	1994–2015	*n* = 2848; 18–84 yrs	Retrospective study design	17.7	−	−	−	−	−	−	−	−	−	−	−	Retrospective study. Screened at urban clinics near Dakar.	C	Data were pooled from multiple prior research studies. BP measured on single visit.
Sierra Leone	Geraedts et al., 2021 [53]	2020	*n* = 1956; 18 yrs+	National household survey	22.0	21.0	22.0	22.7	7.8	14.9	11.1	4.5	6.6	4.5	2.1	2.4	Cross-sectional. National household-based survey. Standardised data.	A	Recall and social acceptability bias for self-reported questions. Lifestyle treatment of hypertension was not considered. BP was measured on single visit.
Togo	−	−	−	−		−	−	−	−	−	−	−	−	−	−	−	–	E	–
***Central Africa***																			
Burundi	Iradukunda et al., 2021 [25]	2018	*n* = 353; 15 yrs+	Physical in-patient screening	16.7	9.3	7.3	−	−	−	−	−	−	−	−	−	Cross-sectional. Screened 2 hospitals / patients randomly selected from a sample of 4380	C	Secondary analyses. Sample size was relatively not large. BP was only reported from 2 hospitals.
Cameroon	Dzudie et al., 2021 [27]	2019	*n* = 30,187; 18 yrs+	MMM	20.8	−	−	29.9	−	−	24.0	−	−	11.2	−	−	Cross-sectional. Screened 15/29 sites.	C	Selection bias (self-selected participants). Insufficient funds to carry out extensive screening. Inability of follow-up (single visit). Non-representative nature of the survey.
Central African Republic	Pengpid et al., 2022 [28]	2017	*n* = 3265; 25–64 yrs	WHO STEPS	42.1	14.5	27.5	30.2	−	−	−	−	−	−	−	−	Subnational cross-sectional. Screened Bangui City and Ombella M’Poko region.	C	Study was only subnationally representative of the general population of this age group.
Chad	−	−	−	−	−	−	−	−	−	−	−	−	−	−	−	−	–	E	–
Comoros	Calas et al., 2022 [29]	2019	*n* = 2602; 18–69 yrs	Unono Wa Maore survey	38.4	38.5	38.3	47.9	38.1	56.2	45.4	49.7	42.9	30.2	29.3	30.8	Cross-sectional. Screened those residing in Mayotte (1/4 islands in Comoros).	C	Study was interrupted by Ramadan; therefore, screening could not be completed. BP measured on a single visit.
Congo, Democratic Republic of the Congo	M’Buyamba-Kabangu et al., 2021 [30]	2019	*n* = 29,857; 18 yrs+	MMM	25.5	−	−	33.1	−	−	23.2	−	−	11.9	−	−	Cross-sectional. Screened in 2 cities.	C	Selection bias (self-selected participants). Insufficient funds to carry out extensive screening. Inability of follow-up (single visit). Non-representative nature of the survey.
Congo, Republic of the Congo	Ellenga-Mbolla et al., 2021 [31]	2019	*n* = 3157; 18 yrs+	MMM	33.5	−	−	42.6	−	−	37.3	−	−	23.3	−	−	Cross-sectional. Screened 15 sites in 5 cities.	C	Selection bias (self-selected participants). Insufficient funds to carry out extensive screening. Inability of follow-up (single visit). Non-representative nature of the survey.
Equatorial Guinea	−	−	−	−	−	−	−	–	–	–	–	–	–	–	–	–	–	E	–
Gabon	Roger et al., 2017 [35]	2013–2015	*n* = 2104; 20–60 yrs	Retrospective study design	13.5	12.8	14.2	−	−	−	−	−	−	−	−	−	Epidemiological study (retrospective and prospective). Screened 2 medical centres in 1 province.	C	BP measurement information lacking. No study limitations noted for this study.
Sao Tome and Principe	−	−	−	−		−	−	−	−	−	−	−	−	−	−	−	–	E	–
***East Africa***																			
Djibouti	−	−	−	−	−	−	−	–	–	–	–	–	–	–	–	–	–	E	–
Eritrea	−	−	−	−	−	−	−	–	–	–	–	–	–	–	–	–	–	E	–
Ethiopia	Tesfa et al., 2021 [34]	2010–2020	*n* = 38,075; 18 yrs+		20.6	−	−	−	−	−	−	−	−	−	−	−	Systematic review including 35 articles to estimate the pooled prevalence. Screened 5 regions in Ethiopia.	D	Search strategy may have missed unpublished articles therefore publication bias could likely have occurred.
Kenya	Ogola et al., 2021 [40]	2019	*n* = 33,992; 18 yrs+	MMM	26.1	−	−	34.5	−	−	31.5	−	−	18.8	−	−	Cross-sectional. Screened 30 sites across 13 counties.	C	Selection bias (self-selected participants). Insufficient funds to carry out extensive screening. Inability of follow-up (single visit). Non-representative nature of the survey.
Madagascar	Manus et al., 2018 [42]	2015–2017	*n* = 513; 18–89 yrs	General Health Survey + BP measures	49.1	20.1	28.5	−	−	−	−	−	−	−	−	−	Cross-sectional. Screened in 1 agricultural village.	C	Biased sampling – unemployed/unhealthy individuals may be more likely to visit the health clinic. Seasonal bias – data was collected during austral winter. BP was measured using Western standards which may not capture the biological factors unique to a non-Western population.
Malawi	Ndhlovhu et al., 2021 [43]	2019	*n* = 9723; 18 yrs+	MMM	26.3	−	−	17.4	−	−	15.2	−	−	7.9	−	−	Cross-sectional. Screened ≈ 37 sites in 3 districts.	C	Selection bias (self-selected participants). Insufficient funds to carry out extensive screening. Inability of follow-up (single visit). Non-representative nature of the survey.
Mauritania	−	−	−	−		−	−	−	−	−	−	−	−	−	−	−	–	E	–
Mauritius	Kowlessur et al., 2021 [45]	2019	*n* = 8262; 18 yrs+	MMM	29.4	−	−	64.7	−	−	60.8	−	−	34.8	−	−	Cross-sectional. Screened across the entire island.	C	Selection bias (self-selected participants). Insufficient funds to carry out extensive screening. Inability of follow-up (single visit). Non-representative nature of the survey.
Rwanda	Nahimana et al., 2018 [50]	2012–2013	*n* = 7116; 15–64 yrs	WHO STEPS	15.4	16.5	14.4	23.0	−	−	−	−	−	−	−	−	Cross-sectional. Secondary analysis of primary data collected from a national population-based study. Data was weighted by sample weights.	A	BP measured on single visit. No further limitations pertaining to BP were noted.
Seychelles	Plumettaz et al., 2020 [52]	2018–2019	*n* = 619; 18–86 yrs	Structured questionnaire	33.8	−	−	−	−	−	22.0	−	−	−	−	−	Cross-sectional. Community-based screening programme in Mahe (main island)	C	Sample size was small. The validity of the hypertension diagnosis in persons in the screening programme treated for hypertension was not assessed. BP measured on single visit and single measure.
Somalia	Nooh et al., 2023 [54]	2022	*n* = 319; 18 yrs+	WHO STEPS	22.6	24.9	19.9	−	−	−	−	−	−	−	−	−	Cross-sectional. Hospital-based screening in Somaliland, northern Somalia	C	Patients were specifically targeted (selection bias). No further limitations pertaining to BP were noted.
South Sudan	Omar et al., 2020 [56]	2018	*n* = 600; 18 yrs+	WHO STEPS	40.8	12.0	28.8	−	−	−	−	−	−	−	−	−	Cross-sectional. Screened in 1 region (Gadarif).	C	More females were screened in this survey than males. No further limitations pertaining to BP were noted.
Sudan	Beheiry et al., 2020 [55]	2018	*n* = 40,779; 18 yrs+	MMM	28.2	−	−	20.7	−	−	18.2	−	−	54.6	−	−	Cross-sectional. Screened 6 Sudanese states.	C	Selection bias (self-selected participants). Insufficient funds to carry out extensive screening. Inability of follow-up (single visit). Non-representative nature of the survey.
Tanzania	Osetinsky et al., 2022 [57]	2020–2021	*n* = 784; 18–89 yrs	Household survey	41.0	−	−	41.0	−	−	20.9	−	−	11.4	−	−	Cross-sectional. Screened in 2 districts.	C	Self-reported NCD diagnosis. BP was measured at single visit.
Uganda	Okello et al., 2020 [19]	2018	*n* = 760; 18 yrs+	WHO STEPS	20.4	−	−	−	−	−	−	−	−	−	−	−	Observational study. Screened 1 semi-urban municipality (Soroti).	C	All participants were of African ancestry and there were no rural communities sampled. Population-based surveys are subject to the healthy volunteer bias.

*n* number of participants, *BP* blood pressure, *LMICs* low-to-middle income countries, *NCD* non-communicable disease, *WHO* STEPS – World Health Organisation STEPwise approach to non-communicable disease risk factor surveillance, *MMM* May Measurement Month, *DHS* Demographic and Health Survey. Prevalence scoring adapted from Micklesfield et al. [61]. Score: A - Published national and regional prevalence data available for this age group; B - Published national and regional prevalence data available, not specific to age group; C - Only regional prevalence data for this age group; D - Only regional prevalence data but not specific to this age group and E - No prevalence data.

## The burden of hypertension in sub-Saharan Africa

### Prevalence, treatment, and control rates

Since 2017, several global, regional, and country-specific studies have confirmed the poor levels of hypertension detection and suboptimal treatment and control in SSA. In this section, we summarise the patterns in detection, treatment, and control rates of hypertension in SSA countries based on reports of the past six years from regional (multinational studies in SSA), national (countrywide representative studies), and location-specific (only limited to a specific area in a particular country) surveys. Some of the national surveys are based on the World Health Organisation (WHO) STEPwise approach to non-communicable disease (NCD) risk factor surveillance (STEPS). Other countries’ data are from the May Measurement Month (MMM) initiative for each country, while some countries have no data for the reporting period.

Schutte et al., [14] reported that SSA results of the 2018–2019 MMM on awareness (40.5%), treatment (32.1%) and control (15.4%) were slightly lower than those reported for the SSA region by the NCD Risk Factor Collaboration. The latter report showed that in women, diagnosis, treatment, and control were 48%, 29 and 13% [2]. A similar trend was evident in men with 34% of diagnosed hypertension and even lower rates of treatment (22%) and control (9%). The analysis included nationally representative studies and statistical analysis included weighting and adjustments for complex study designs, which is not the case for MMM data. [2].

**Fig. 1:**
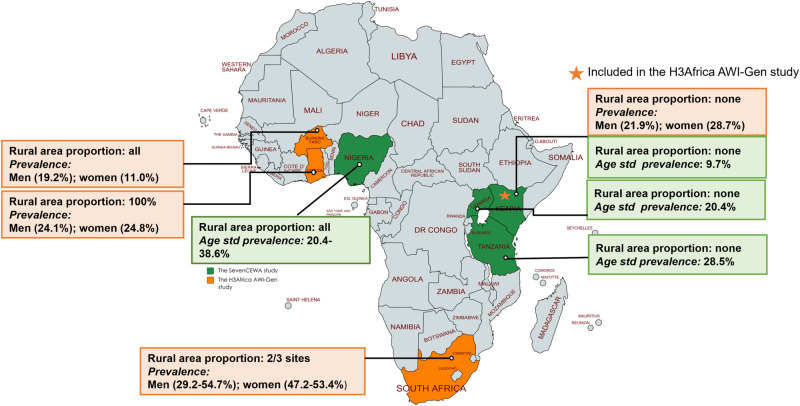
Inclusion of rural areas in multinational studies assessing hypertension prevalence in sub-Saharan Africa. This figure depicts the two major recent multinational studies (H3Africa AWI-Gen; SevenCEWA) that presented data on hypertension prevalence, awareness, treatment, and control in rural and urban areas of sub-Saharan Africa. The green and orange colors show the included countries, while grey represents countries not included in the two studies. The orange star indicates that the country was included in both studies. H3Africa Human Heredity and Health in Africa, SevenCEWA seven communities in East and West Africa.

One of the most recent multinational studies that applied standardised methods across all sites is the seven communities in East and West Africa (SevenCEWA) study [19]. This cross-sectional study included 3549 participants aged 18 years and older from seven communities in Kenya, Nigeria, Tanzania, and Uganda. The mean age was 39.7 years (SD, 15.4), and about a quarter (25.4%) of the study population had hypertension, with 57.2% awareness rate, just over half of these individuals being treated, and close to 47.3% achieving BP control targets (Fig. 1). Earlier on, the Human Heredity and Health in Africa (H3Africa) AWI-Gen study reported a higher prevalence of 33% in 10696 participants aged between 40 and 60 years (mean age 49.9, SD 0.06) from Burkina Faso, Ghana, Kenya, and South Africa [20]. In this study, overall awareness was at 47.7%, and awareness, treatment, and control rates differed according to study sites between and within countries, with the highest prevalence in South Africa (41.6%–54.1%) and lowest in Burkina Faso (15%), which is closer to the values recently published in national surveys in both countries although using different study designs and data collection methods [7, 21]. In contrast, results from the sites in Kenya included in both the SevenCEWA and H3Africa AWI-Gen are not within the same range (Fig. 1). As indicated in most of the surveys in Table 1, the disparities may be due to purposive and convenience sampling and data collection methods. This underscores the need for standardised methods and inclusion of nationally representative populations.

**Fig. 2:**
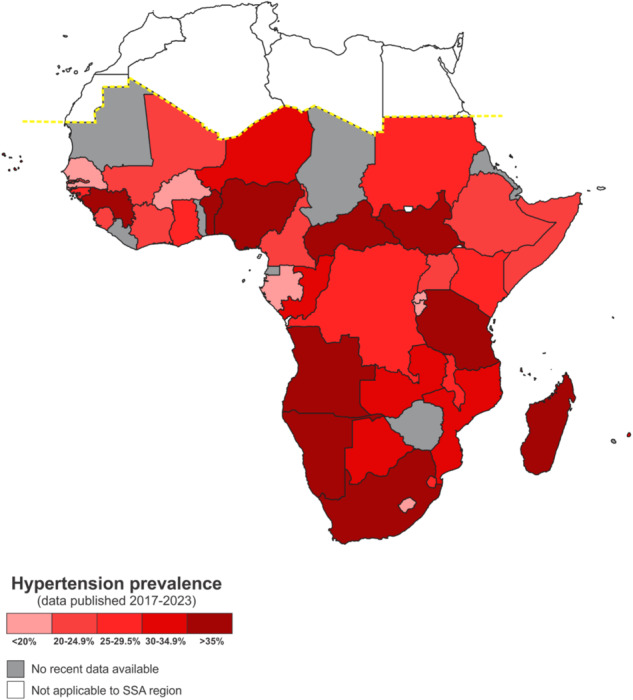
Recent multinational and country-specific studies assessing hypertension prevalence, treatment and control in sub-Saharan Africa. This figure depicts recent (2017–2023) prevalence, treatment and control rates reported in nationwide and regional (within country) surveys in sub-Saharan Africa. Where multiple surveys are available for the 2017–2023 period, the most recent one is presented. SSA sub-Saharan Africa.

Recent (2017–2023) prevalence, treatment and control rates reported in nationwide and regional (within country) surveys are summarised in Fig. 2 and Table 1 [7, 19, 21–60]. Where multiple surveys are available for the 2017–2023 period, the most recent one is presented, and the prevalence scoring was adapted from Micklesfield et al., [61]. These surveys have mostly indicated that the prevalence of hypertension and comorbidities is increasing in rural areas, however, there is variation across the care cascade [7, 11, 20, 62]. Some of the limitations in reported surveys are reflected in the heterogeneity of included study populations. First is the age range, with the lowest age of inclusion being 15 years while the inclusion for other studies started as high as 40 years of age [7, 20, 47, 63]. Differences between men and women are not always presented across the care cascade (Table 1), and some studies included only urban or rural dwellings such as study sites in Kenya (only urban) and Nigeria (all rural) in the H3Africa study [20] (Fig. 1). Urbanisation and the sociocultural transition associated with a westernised lifestyle in rural areas have been echoed by several studies as the main driver for the increase in the prevalence of hypertension in rural areas to a level comparable to the prevalence in urban populations [7, 11, 12]. Additionally, rural areas have higher odds of undiagnosed hypertension due to the reach of healthcare services, among other factors [53].

### Management of hypertension in sub-Saharan Africa

Barriers to the control of hypertension in SSA include individual-and system-level aspects. At an individual level these include, health literacy, especially around hypertension and CVD and socioeconomic factors that affect the affordability of health insurance and medicines (out-of-pocket costs) and distance to healthcare facilities [19, 64–66]. System-related barriers include the availability of medicines, poor access to healthcare facilities and limited workforce capacity and knowledge of the healthcare workers as well as political will in implementing interventions that have been shown to work elsewhere in the management of hypertension [14, 63–65]. It was recently reported in a Soweto population that the poor quality of PHC services such as unpleasant interactions of healthcare providers with patients may serve as some of the deterrents for visiting PHCs [67]. As a result, tasking-sharing is one of the most relevant approaches for improving the management of hypertension in resource-limited contexts such as in SSA countries [4, 67, 68]. Training of role model patients as facilitators for support groups and community health workers (CHWs) to provide clinical and nonclinical support have the potential to counteract some of the individual and system barriers mentioned above.

Table 2 summarises different strategies that tested the role of task-shifting in the form of allocation of some of the responsibilities in hypertension management to role model patients and/or CHWs. Overall, depending on the design and implementation strategies, most forms of task-shifting improved linkage to health services and adherence to medication [69–71], with inconsistent results on the effect on BP control over time. One of the relevant recent findings come from the HealthRise programme, which was implemented in Brazil, India, South Africa, and the United States of America [65]. South African sites were amongst the most challenging, which included two rural areas in KwaZulu Natal and the Northern Cape provinces. Main limitations that may explain the unfavourable results from the South African sites are the short implementation period, socioeconomic and demographic factors such as residing in a rural area, low education, and literacy levels in patients, and the high unemployment rate as well as health system-related barriers [65]. Additional limitations of task-shifting studies in SSA have been reported in a recent scoping review and framework for designing chronic services [72]. These include attrition rates, quality of studies, parallel as opposed to integrated models, and lack of comparison groups.

**Table 2:** Community-based strategies for management of hypertension in sub-Saharan Africa.

Country	Author, published (year)	Study sample, *n* + age range (years)	Study design	Study setting	Intervention	Blood pressure outcomes
Nigeria	Isiguzo et al., 2022 [70]	104, 33–85	Mixed methods	PHC facilities	‐ Assessed the impact of adherence clubs on hypertension control and medication adherence. ‐ One role-model patient served as club facilitator. ‐ PHC nurses and CHWs served as point of contact for clinical support.	‐ Clinically meaningful improvement (15.5%, *p* < 0.0001) in self-reported medication adherence. ‐ Reduction in mean systolic (by 13.0 mmHg, *p* < 0.0001) and diastolic (by 3.6 mmHg, *p* = 0.02) BP
South Africa	Ameh et al., 2020 [69]	878, ≥ 18	Mixed methods	PHC Facilities	‐ Assessed the effectiveness of the ICDM model in controlling CD4 cell counts and blood pressure of HIV and hypertension patients. ‐ PHC facilities divided into pilot (intervention) and control (usual care).	‐ Intervention facilities had a 1.0% greater chance of BP control than the comparison facilities, (*p* = 0.002).
Multinational-^a^South Africa	Flor et al., 2020 [65]	150, 15–70	Mixed methods quasi-experimental	PHC facilities and community-based care givers	‐ Assessed impact of the Health Rise programme on screening, diagnosis, management, and control of hypertension and diabetes among underserved communities. ‐ Included technology for care coordination, workforce development, clinical and non- clinical patient support. ‐ Training of CHWs, CCGSs and healthcare workers in KZN and the Northern Cape to screen for hypertension and diabetes. ‐ Support/adherence groups to provide health education and importance of adherence.	‐ No detectable differences in attendance of facilities between HealthRise interventions and usual facilities, *p* > 0.05 ‐ Only cross-sectional patient data available at endline ‐ Unable to assess changes along the care cascade from baseline to endline among HealthRise implementation and comparison areas.
Kenya	Vedanthan et al,. 2019 [71]	1460, ≥ 18	Cluster randomised trial	Healthcare facilities and Community units	‐ Assessed the impact of CHWs on linkage to hypertension care and BP reduction among individuals with elevated BP. ‐ Compared three groups including the usual care, paper-based tools and smart phone. ‐ CHWs trained in tailored behavioural communication	All groups experienced a decline in blood pressure, but not a statistically significant reduction. ‐ *Usual care:* –9.7 ± 25.1 ‐ *Paper-based tools*: –8.4 ± 24.0 ‐ *Smartphones*: –13.1 ± 20.5

^a^Conducted in Brazil, India, South Africa and USA. Results in the table will focus only on South African sites.

*ICDM* integrated chronic disease management, *HIV* human immunodeficiency virus, *PHC* primary healthcare, *BP* blood pressure, *CHWs* community health workers, *KZN* Kwa-Zulu Natal, *CCGs* community care givers.

The integration of human immunodeficiency virus/acquired immunodeficiency syndrome (HIV/AIDS) and hypertension care in another rural setting in the Mpumalanga province demonstrated that the integration model has the potential to improve hypertension management, even though the treatment targets for HIV/AIDS were met more frequently than BP treatment targets [69]. In Kenya, a comparison of different levels of care showed that shifting some of the responsibilities from clinicians and nurses to CHWs by incorporating behaviour change communication using either paper-based tools or smartphones resulted in improved linkage to care and some non-significant decline in BP [71]. In contrast, the significant decline in both systolic and diastolic BP in the CLUBMEDS intervention in Nigeria was attributed to, among others, understanding and acceptability of the intervention by patients which improved adherence, similar to application of the same intervention to HIV/AIDS management approaches, which was the basis of this strategy in hypertension management [70]. Although the current studies had limitations, newer feasibility studies for community-based interventions for the management of hypertension offer promise for the knowledge base on the utility and sustainability of community-based interventions to improve care for people living with hypertension in SSA [73, 74]. On pharmacological interventions, optimal medical therapies need to be further investigated. The CREOLE trial has reported that combination of a diuretic with a calcium channel blocker provides efficient BP control [75] and further studies with representation from various regions in Africa are urgently needed to address the current treatment gaps.

## Recent advances in hypertension in sub-Saharan Africa

### Risk factors

The main reported drivers of hypertension prevalence and disparities between and within countries in SSA are diet [15, 59, 76], increased adiposity and underweight [47, 56, 77], ageing [12, 19, 56, 62], level of education, and/or income as well as psychosocial factors [12, 15, 19, 47]. These factors are likely influenced by culture and religious practices as reported in recent surveys from Western, Eastern, and Southern African countries [63, 64, 78]. In multinational and national studies, the prevalence of hypertension tends to still be higher in urban than in rural areas within some countries such as South Africa and Namibia [7, 8, 47, 79], although the gap is closing. This is reflected in the most common modifiable risk factors being shared between urban and rural areas as reported in Ghana, Nigeria, South Africa, Kenya, and Tanzania [11, 12, 57, 79]. The rate at which different areas undergo the epidemiological transition may be one of the reasons for the inconsistencies in rural versus urban areas gaps within countries [79, 80].

Early life programming due to adverse maternal risk factors before conception and during pregnancy elevates the risk for premature onset of CVDs [81, 82]. Briefly, intrauterine and early life exposure to risk factors such as maternal smoking, alcohol consumption, psychological stress, perinatal complications, malnutrition, low socioeconomic status and lack of prenatal care can directly impact the offspring’s risk for hypertension and CVD [81–85]. Below we discuss the risk factors highlighted from SSA populations in the past six years.

#### Nutrition

Unhealthy dietary habits associated with hypertension in SSA are characterised by a high intake of food rich in sodium and fewer fruits and vegetables as primary sources of potassium [13, 64, 76, 86–89]. Salt intake is independently associated with prevalent hypertension in several countries. A study in Benin reported high salt intake as an independent predictor of hypertension and noted sex differences, with women having a higher use and intake than men (63.3% versus 49.7%) [63]. Data from Zimbabwe showed that individuals with hypertension who did not put additional salt in food during mealtime had a 40% reduction in the odds of uncontrolled hypertension [59], consistent with previous results from Ghana [90].

In rural Zambia, the median intake of both sodium and fatty acids was found to be below the recommended level and not associated with hypertension [91]. In rural Ghana, sodium, potassium and energy intake were lower in the local population as compared to levels in Ghanaians living in Europe, underscoring the detrimental role of acculturation to a western lifestyle [9]. In South Africa, before the implementation of the salt legislation in 2016, 69% of adults exceeded the > 5 g salt/day cut-off recommended by the WHO, while a startling 91% did not meet the daily requirement for potassium intake [13]. In this WHO-SAGE study, the ratio of sodium to potassium was associated with a steeper regression slope for the increase in BP with age. Recent preliminary data shows the impact of salt legislation on salt intake with a reduction of approximately 1.2 g salt per day over 4.56 years, particularly in Black South Africans with low socioeconomic status, suggesting greater potential for hypertension risk reduction in high-risk African populations [92].

Socioeconomic inequality is a critical driver for poor nutrition. In East Africa, 26–38% of children under the age of five are undernourished [93]. Malnutrition remains a critical area for intervention in SSA, with stunting in children decreasing by 8% between 2000 and 2017 [94], while the number of affected children increased due to population growth. Communities especially in Nigeria, Ethiopia, Somalia, and Kenya are mostly affected by stunting, which directly impacts on paediatric BP percentiles for detecting hypertension [93].

#### Sedentary lifestyle and obesity

The prevalence of physical inactivity is of grave concern with rates in adults above 22% and in adolescents more than 85% [95]. Undoubtably, a lack of physical activity and increased sedentary behaviour contribute to the rise in obesity, a known leading risk factor for CVD. Obesity prevalence in Africa ranges between 4.5 and 32.5% [96], while the overweight/obesity group hypertension prevalence in children and adolescents is approximately 18.5% [97]. In South Africa and Nigeria, two of the SSA countries with the highest prevalence of hypertension based on different studies, a sedentary lifestyle associated with a move from rural to urban or westernised life has been cited as the major contributor to the high prevalence of hypertension [20]. A recent study in rural Zambia shows that the role of obesity in hypertension prevalence is not unique to urban areas but is a growing concern even in rural-dwelling postmenopausal women [91]. In the H3Africa AWI-Gen study, hypertension was driven by obesity and physical inactivity in women aged 35–44 years residing in an informal settlement in Nairobi [20], similar to women in Benin [63]. In the multicountry SevenCEWA study, being overweight/obese was associated with higher mean systolic BP [19], while in Namibia, although overweight and obesity were more common in women than in men, the odds of hypertension were higher in both overweight/obese men and women [47].

#### Alcohol and smoking

Inconsistent data have been reported on the role of tobacco and alcohol use as predictors of hypertension in SSA. In countries such as South Africa and Ghana, these two risk factors are associated with hypertension prevalence and contribute to its variation between population groups [7, 9]. Of interest, in areas such as Benin, tobacco and alcohol use were not influential to the prevalence of hypertension, possibly due to the low use, which is inconsistent with data from other SSA countries [63]. Tobacco and alcohol use’s weak or lack of association with hypertension prevalence may be due to under-reporting, or limited use in some regions as reported in Sudan and Uganda [56, 98]. Additional explanations for lack of associations between alcohol use and hypertension are differences in settings and methods used to assess alcohol intake, such as subjective methods which are associated with recall bias [56]. Although alcohol use and smoking were among the main contributors to the high hypertension prevalence in a nationwide survey in Zimbabwe, recent data from a rural community found opposing results and suggest low income as one of the reasons for the low use of alcohol and tobacco [64].

#### Demographic factors

In the H3Africa AWI-Gen study, which included East, West and Southern African countries, one of the most common risk factors for hypertension prevalence (28.7%) was age [20]. In the SevenCEWA study, covering East and West Africa, a 10-year increase in age was associated with increased odds of prevalent hypertension (adjusted Odds Ratio 1.4, 95%Ci 1.4–1.5) [19]. Increasing age is considered a risk factor for hypertension as observed in most surveys [19, 63]. Certain age groups seem to be at an increased risk of prevalent hypertension based on risk profile [12], underdiagnosis and adherence to treatment [12]. Simultaneously, other studies have found age to not be associated with the hypertension prevalence and management [62, 63, 99]. The association of age with hypertension risk, generally reflect the effect of the cumulative exposure to hypertension risk factors over the life course.

When investigating the role of socioeconomic factors, having some form of education as opposed to having no education at all was associated with a lower prevalence of hypertension, particularly in women, whereas attainment of primary-level education was associated with lower odds of awareness. In Namibia, the odds of hypertension were low for women with higher levels of education [47]. Health insurance was associated with lower hypertension prevalence and increased likelihood of being treated among women [19]. A recent study in KwaZulu Natal showed that Black South Africans living in under-resourced areas had higher odds of being hypertensive and less likelihood of BP control [79]. A similar observation was found in Sierra Leone, where male sex, rural location and age were associated with higher odds of undiagnosed hypertension [53], while detection and treatment levels are usually higher in urban versus rural areas as reported in nationwide survey conducted in Nigeria [11]. A recent report of the South African National Health and Nutrition Examination Survey has reiterated older age, male sex and Black African ethnicity as the factors contributing the most to the hypertension burden and requires prioritisation in terms of preventative approaches [7].

Hypertension risk factors, detection, treatment and control rates discussed in this review are highly influenced by cultural practices as reported from Western, Eastern, and Southern African countries [63, 64, 78]. A study in a disadvantaged community in Southern Zimbabwe showed that participants who believed in herbal medicines (50.7%) and those who used traditional medicines (14.5%) were less likely to have knowledge about hypertension as compared to participants who did not believe in or use traditional medicines [64]. This impact of traditional and religious beliefs on detection and optimal treatment of hypertension is widespread in SSA [53, 56, 62, 78] and calls for research that is informed by stakeholder engagement from the formative stages to better respond to the diverse profiles of hypertension and CVD in SSA.

### Hypertension phenotypes and underlying mechanisms

Most of the studies presented in the last few decades on risk factors for hypertension and pathophysiological mechanisms linking these risk factors to hypertension have focused on general populations, older individuals, patients already living with hypertension and/or populations with comorbidities [19, 100–102]. Some of the recent studies in the SSA setting that provide insights into the early alterations associated with BP elevation and CVD risk include the Exercise, Arterial Modulation and Nutrition in Youth South Africa (ExAMIN Youth SA) study [103] and the African Prospective study on the Early Detection and Identification of Cardiovascular disease and Hypertension (African-PREDICT) [104]. Earlier longitudinal studies such as the Birth to Twenty (Thirty) cohort study [84] and the Ellisras longitudinal study [105] had already established the sociodemographic and dietary patterns associated with the increase in hypertension prevalence when observing populations from birth until adulthood and intergenerational factors.

#### Sodium and potassium handling

A review of the literature currently still highlights sodium intake and handling as one of the main factors explaining the hypertension phenotypes commonly observed in populations of African ancestry [13, 106, 107]. Sodium excretion as a measure of salt intake was positively associated with left ventricular mass index, a marker of cardiac remodelling in participants with masked hypertension [108]. In this South African cohort, approximately half of the study population comprised of Black Africans. Salt intake was further linked to cardiovascular abnormalities associated with BP elevation such as arterial stiffness in Black Africans only [109]. Interestingly, associations between salt and potassium intake and mechanisms such as inflammation did not show any link to Black African ancestry in the same cohort [110]. In the Ellisras study, in rural children aged 5–13 years, sodium intake was positively associated with systolic and diastolic BP, while an inverse correlation was observed between potassium and systolic BP [87].

The lower activity of the renin angiotensin aldosterone system (RAAS) in Black populations, especially in relation to volume expansion and high salt intake is well-known as a determinant of hypertension phenotypes in Africans [16, 111, 112]. Recently, the role of potassium in modulating sodium handling and its effects on BP regulation has become a topic of interest. In the African-PREDICT study, components of the RAAS measured by the RAS Fingerprint^®^ were markedly lower in healthy Black men and women as compared to their White counterparts [113]. The incorporation of potassium, in the form of a sodium-to-potassium ratio as opposed to using only sodium, diminished the differences in plasma renin activity (PRA) between Black South Africans and their White counterparts in the group with the lowest levels of PRA [113]. In that same group, aldosterone levels predicted a percentage increase in central systolic BP over approximately 4.5 years in young Black Africans. Sex differences were also observed in this group with measures of aldosterone excess [aldosterone-to-renin ratio (ARR), aldosterone-to-angiotensin II ratio AA2R] found to positively associate with central and peripheral BP only in young Black African women and only with peripheral BP in their male counterparts [114].

Other components of the RAAS cascade seem to not play a role in BP and early cardiovascular alterations associated with hypertension in Black Africans. Among these is the prorenin receptor, first discovered in 2002 [115] and was measured in a soluble form in the African-PREDICT study. This biomarker was high in White participants as compared to Black South Africans and adversely associated with left ventricular function in White women and not in any of the Black African groups [116]. Although this receptor is new and involved in profibrotic and proinflammatory pathways as well as sodium retention via the actions of angiotensin II [115], its relevance in hypertension and cardiovascular risk assessment in Black Africans is not clear at present. Further prospective studies might shed more light on this. This observation, among others, supports the need for knowledge generation to inform not only the public health response to the high hypertension prevalence and suboptimal treatment and control levels but the understanding of the pathways leading to BP elevation which may facilitate pharmacological and non-pharmacological interventions tailored for Black Africans.

#### Volume loading

Although it was well-known for several decades that the low renin phenotype frequently observed in populations of African ancestry was mainly attributable to disproportionate sodium retention and volume expansion [111, 112], recent data add more explanations to the role of volume regulation in hypertension in Africans. A community-based study in South Africa has demonstrated that systemic flow as indicated by stroke volume, cardiac output and peak aortic flow are primary determinants of hypertension across the adult age spectrum [117]. It was further shown that relationships between salt intake as indicated by sodium-to-potassium ratio and BP can be explained by aortic characteristic impedance and not systemic flow [118]. In turn, age-related elevation in systemic flow emerged as the predictor of sodium excretion and kidney function [119]. These observations suggest some level of independence between salt-sensitivity and renal mechanisms associated with hypertension development and underscores the need for therapeutic interventions for hypertension in Africans not accounted for by current treatment regimens [117–119].

#### Salt sensitivity

The concept of salt sensitivity has always been linked to the suppression of renin in populations of African ancestry due to sodium and water retention [111, 120]. Salt sensitivity is associated with important and common risk factors as well as pathways linked to hypertension such as diet and obesity [112]. A study in normotensive young South African men and women found a positive association between salt intake and body surface area as a measure of adiposity and not with traditional measures such as body mass index [121], confirming the potential role of skin in sodium storage and salt handling [122]. In the same study, potassium, an important determinant of salt sensitivity, was shown to have a protective effect at low sodium levels, however, this benefit was not evident in Black participants [110]. Of interest, in White participants of this study, the relationship between high salt intake and BP was related to metabolomic changes assessed using the targeted metabolomics technique [123]. Furthermore, marinobufagenin, a marker of salt sensitivity was associated with both increased left ventricular mass in obesity and influenced microvascular function in non-dippers in the same study [124]. This marker further showed a unique ethnicity related association with BP in young Black South African women [125]. When looking at data from other SSA regions, high sodium intake was recently associated with a non-dipping pattern in the CREOLE trial, which included multiple sites in six SSA countries (Cameroon, Kenya, Mozambique, Nigeria, South Africa and Uganda) [126]. The non-dipping pattern had a prevalence of 78% in individuals with uncontrolled hypertension, further supporting the need for tailored therapeutic interventions in the African context [126].

#### Endothelial function

Alpha-adrenergic receptor function has long been linked to raised BP in individuals of African ancestry and forms some of the basis for calcium channel blockers as one of the first-choice therapy in populations of African descent [127, 128]. Recent data from individuals of different age groups and health status continue to support the central role of endothelial integrity and function in BP regulation, kidney function and hypertension in SSA. In Black South African adults, nitric oxide synthesis was inversely associated with central systolic BP and urinary albumin-to-creatinine ratio as a marker of kidney function and endothelial dysfunction [129, 130]. Furthermore, urinary alpha-1-microglobulin, a marker of renal tubular function was associated with odds of having elevated BP by 28% in Black prepubescent children [131]. In addition, the role of nitric oxide and oxidative stress in BP control was more evident in boys and men [132], a group known to have a higher risk for hypertension and CVD.

#### Human immunodeficiency virus

With SSA, specifically South Africa as the epicentre of the HIV epidemic, the nexus between communicable and non-communicable diseases, especially HIV and CVD is of public health concern. Persistent low-grade inflammation, despite treatment in some cases is one of the pathways potentially linking HIV and hypertension risk [133]. However, recent reports from SSA have consistently shown that HIV is not associated with an increase in BP or the risk for hypertension and CVD compared to those without [134–137]. A study in Uganda presented a lower prevalence of hypertension in people living with HIV and HIV status was associated with lower odds of hypertension, elevated systolic and diastolic BP [136]. CD4 cell count, viral load and antiretroviral therapy (ART) did not seem to influence the odds of hypertension. This is consistent with findings from different areas in South Africa showing that individuals living with HIV have lower BP profiles than their HIV-negative counterparts [134, 135]. Along the same lines, a study in South Africa showed that low-level viremia in patients on ART was not associated with cardiovascular risk [138]. Generally, data from different cohorts confirm that people living with HIV don’t present with a worse cardiovascular profile as compared to controls. Perhaps a different approach to the investigations and longer follow-up periods might provide unknown explanations of the current profiles.

## Implications for knowledge gaps and priorities

### Knowledge generation and application

Knowledge generation in terms of understanding the pathways and factors leading to hypertension is equally important to identify areas where early interventions can reduce the risk for hypertension and hypertension-mediated target organ damage. Some of the mechanisms associated with an increase in BP in Black Africans suggest the need for alternative therapy to improve control of BP. Mechanistic research into risk factors and biomarkers in Africans has the potential to inform personalised medicine for the African context. Capacity building should therefore include support for research and innovation that includes the generation of new knowledge, translation and application that will have a wider and more sustainable impact. Evidence on hypertension mediated organ damage and severity of hypertension are, likewise, lacking. Further epidemiological studies need to address this discrepancy in order to stratify patients according to risk and further optimise medical care.

Another crucial gap is the attention to risk factors and hypertension development in children, which is likely to persist into adulthood and contribute to the burden on healthcare systems. Childhood BP nomograms are lacking across Africa and the rates of hypertension are based on guidelines developed in countries with the lowest to no number of children from African ancestry [139]. The recent studies across Africa show little to no detail in reporting BP specific methodology and practices. No recent data informing the use or effectiveness of antihypertensive agents in African children or adolescents are available. While childhood hypertension is on the rise, data from Africa remains vastly under-represented [139].

### Representative studies

As a follow-up to the 2017 roadmap to achieve 25% hypertension control in Africa [4], Owolabi et al. published strategies [Innovative Epidemiology and a Vibrant Ecosystem (ACHIEVE)] to accelerate hypertension control in Africa [68]. These strategies are reflected in the actions outlined in the Call to Action to improve awareness, treatment, and control of Hypertension in Africa, from the World Hypertension League [140]. In line with these initiatives, our current review highlights the need for standardised methods and coordinated implementation of proposed actions across SSA, while taking into consideration the vast diversity in terms of sociodemographic and economic factors. Recent data published on the prevalence, treatment, and control of hypertension in SSA points to an improvement in reporting from most countries, although not always representative of nationwide conditions and mostly from the MMM global screening campaign. This underscores the need for more nationwide BP screening and follow-up to accurately estimate the burden of hypertension as this will inform adequate and evidence-based interventions.

### Prevention

Nationwide preventative strategies should target modifable risk factors that were commonly reported in the review period. These include obesity and undernutrition [47, 56, 77], sedentary lifestyle [95, 96], unhealthy diet [15, 59, 76] characterised by high intake of sugar, sodium and fat, alcohol consumption [7, 9, 64] and smoking [7, 9]. Some countries in SSA have already indicated the importance of stakeholder engagement and political will in the implementation of the salt intake reduction [141] and trans fats elimination [142, 143] legislations. There are still gaps on actions to ensure monitoring and evaluation to achieve the desired benefit of the policies on hypertension and CVD prevention. Screening for hypertension and co-morbidities, health education and addressing socioeconomic inequalities is crucial for both prevention and management of hypertension and associated complications [12, 15, 19, 47]. More efforts should be made to foster collaboration between government, researchers and communities to facilitate implementation of strategies to improve awareness of risk factors for hypertension and CVD across the life course.

### Contextual differences

Most importantly, in the context of social determinants of health, the variation in cultural aspects, especially when accompanied by poverty has a significant impact on detection, treatment and control of hypertension throughout the SSA region. These issues have a prominent effect in rural settings and are exacerbated by limited knowledge and understanding of chronic conditions such as hypertension and poor access to standard healthcare [14, 53, 62]. In terms of risk factors, cultural and religious beliefs influence research on modifiable risk factors such as alcohol consumption and smoking, resulting in underreporting [14, 56, 98] and subsequently the political will for policy to address these risk factors [14]. At health systems level, limited resources including staff, antihypertensive medication and BP measuring devices serve as a major barrier to screening, diagnosis, treatment and control of hypertension [144]. The disparities in resources are also evident within countries where some communities are underserved compared to others when comparing urban and rural areas [65, 79]. This emphasises the need for planned actions to be implemented equitably within and between countries and regions.

## Conclusion

The escalating prevalence of hypertension, alongside suboptimal treatment and poor control rates in SSA is clearly a major public health concern. Risk factors including unhealthy lifestyle, obesity, unfavourable socioeconomic conditions and disparities attributed to demographic factors such as age, sex and area of residence are the main drivers of the high prevalence. These are further linked to some individual-level barriers to hypertension detection, treatment and control, which are exacerbated by system related barriers to efficient management of hypertension. Therefore, contextualised interventions to mitigate risk factors across the life course and improve hypertension awareness, treatment and control by linking knowledge generation with application are urgently needed. These efforts have the potential to prevent the dire health and economic effects of hypertension in SSA, particularly on the already strained healthcare systems and well-known socioeconomic inequalities.

## Supplementary information

Supplementary Table 1
