# Supplementary material for: Hypertension in sub-Saharan Africa: the current profile, recent advances, gaps, and priorities
Source: J Hum Hypertens. 2024 May 2;39(2):95–110. doi: 10.1038/s41371-024-00913-6 (PMC11867975; doi:10.1038/s41371-024-00913-6)
Supplement: Supplementary file 1 — Supplementary Table 1 [file 41371_2024_913_MOESM1_ESM.docx]

**Supplementary Table 1: Search strategy adopted for the literature review**

| **Keyword search for population** |
| --- |
| ((((("hypertension"[MeSH Terms] OR "hypertension"[All Fields]) OR ("blood pressure"[MeSH Terms] OR ("blood"[All Fields] AND "pressure"[All Fields]) OR "blood pressure"[All Fields] OR "blood pressure determination"[MeSH Terms] OR ("blood"[All Fields] AND "pressure"[All Fields] AND "determination"[All Fields]) OR "blood pressure determination"[All Fields] OR "arterial pressure"[MeSH Terms] OR ("arterial"[All Fields] AND "pressure"[All Fields]) OR "arterial pressure"[All Fields]) OR ("isolated systolic hypertension"[MeSH Terms] OR ("isolated"[All Fields] AND "systolic"[All Fields] AND "hypertension"[All Fields]) OR "isolated systolic hypertension"[All Fields] OR ("systolic"[All Fields] AND "hypertension"[All Fields]) OR "systolic hypertension"[All Fields]) OR (("diastole"[MeSH Terms] OR "diastole"[All Fields] OR "diastolic"[All Fields]) AND ("hypertension"[MeSH Terms] OR "hypertension"[All Fields])) OR ("blood pressure"[MeSH Terms] OR ("blood"[All Fields] AND "pressure"[All Fields]) OR "blood pressure"[All Fields] OR ("systolic"[All Fields] AND "blood"[All Fields] AND "pressure"[All Fields]) OR "systolic blood pressure"[All Fields]) OR ("blood pressure"[MeSH Terms] OR ("blood"[All Fields] AND "pressure"[All Fields]) OR "blood pressure"[All Fields] OR ("diastolic"[All Fields] AND "blood"[All Fields] AND "pressure"[All Fields]) OR "diastolic blood pressure"[All Fields])) AND ((("federal government"[MeSH Terms] OR ("federal"[All Fields] AND "government"[All Fields]) OR "federal government"[All Fields] OR "national"[All Fields]) AND ("epidemiology"[Subheading] OR "epidemiology"[All Fields] OR "prevalence"[All Fields] OR "prevalence"[MeSH Terms])) OR (("residence characteristics"[MeSH Terms] OR ("residence"[All Fields] AND "characteristics"[All Fields]) OR "residence characteristics"[All Fields] OR "community"[All Fields]) AND based[All Fields] AND study[All Fields]) OR (("population"[MeSH Terms] OR "population"[All Fields] OR "population groups"[MeSH Terms] OR ("population"[All Fields] AND "groups"[All Fields  ]) OR "population groups"[All Fields]) AND based[All Fields] AND study[All Fields]))) |
| **Keyword search for intervention** |
| ((((“Hypertension”)) AND (Hypertension Management[MeSH Terms])) OR (Hypertension Treatment[MeSH Terms])) AND (Strategies[MeSH Terms])) |
| **Keyword search for hypertension development** |
| ((((“Hypertension”)) AND (Hypertension Risk factors[MeSH Terms])) OR (Hypertension underlying factors[MeSH Terms])) OR (Hypertension Underlying mechanism[MeSH Terms])). |
| **Keyword search for outcome** |
| ((((“Hypertension”)) AND (Hypertension Burden[MeSH Terms])) OR (Hypertension Incidence[MeSH Terms])) OR (Hypertension Prevalence[MeSH Terms])) |
| **Keyword search for study settings** |
| ((("angola"[MeSH Terms] OR "angola"[All Fields]) OR ("benin"[MeSH Terms] OR "benin"[All Fields]) OR ("botswana"[MeSH Terms] OR "botswana"[All Fields]) OR ("burkina faso"[MeSH Terms] OR ("burkina"[All Fields] AND "faso"[All Fields]) OR "burkina faso"[All Fields]) OR ("burundi"[MeSH Terms] OR "burundi"[All Fields]) OR ("cameroon"[MeSH Terms] OR "cameroon"[All Fields]) OR ("cabo verde"[MeSH Terms] OR ("cabo"[All Fields] AND "verde"[All Fields]) OR "cabo verde"[All Fields]) OR (("cabo verde"[MeSH Terms] OR ("cabo"[All Fields] AND "verde"[All Fields]) OR "cabo verde"[All Fields] OR ("cape"[All Fields] AND "verde"[All Fields]) OR "cape verde"[All Fields]) AND ("central african republic"[MeSH Terms] OR ("central"[All Fields] AND "african"[All Fields] AND "republic"[All Fields]) OR "central african republic"[All Fields])) OR ("chad"[MeSH Terms] OR "chad"[All Fields]) OR ("comoros"[MeSH Terms] OR "comoros"[All Fields]) OR ("congo"[MeSH Terms] OR "congo"[All Fields]) OR (democratic[All Fields] AND republic[All Fields] AND ("congo"[MeSH Terms] OR "congo"[All Fields])) OR zairi[All Fields] OR ("cote d'ivoire"[MeSH Terms] OR ("cote"[All Fields] AND "d'ivoire"[All Fields]) OR "cote d'ivoire"[All Fields]) OR ("djibouti"[MeSH Terms] OR "djibouti"[All Fields]) OR ("equatorial guinea"[MeSH Terms] OR ("equatorial"[All Fields] AND "guinea"[All Fields]) OR "equatorial guinea"[All Fields]) OR ("eritrea"[MeSH Terms] OR "eritrea"[All Fields]) OR ("ethiopia"[MeSH Terms] OR "ethiopia"[All Fields]) OR gaben[All Fields] OR ("gambia"[MeSH Terms] OR "gambia"[All Fields]) OR ("ghana"[MeSH Terms] OR "ghana"[All Fields]) OR ("guinea"[MeSH Terms] OR "guinea"[All Fields]) OR ("guinea-bissau"[MeSH Terms] OR "guinea-bissau"[All Fields] OR ("guinea"[All Fields] AND "bissau"[All Fields]) OR "guinea bissau"[All Fields]) OR ("kenya"[MeSH Terms] OR "kenya"[All Fields]) OR ("lesotho"[MeSH Terms] OR "lesotho"[All Fields]) OR ("liberia"[MeSH Terms] OR "liberia"[All Fields]) OR ("madagascar"[MeSH Terms] OR "madagascar"[All Fields]) OR ("malawi"[MeSH Terms] OR "malawi"[All Fields]) OR ("mali"[MeSH Terms] OR "mali"[All Fields]) OR ("mauritania"[MeSH Terms] OR "mauritania"[All Fields]) OR ("mauritius"[MeSH Terms] OR "mauritius"[All Fields]) OR ("mozambique"[MeSH Terms] OR "mozambique"[All Fields]) OR ("namibia"[MeSH Terms] OR "namibia"[All Fields]) OR ("niger"[MeSH Terms] OR "niger"[All Fields]) OR ("nigeria"[MeSH Terms] OR "nigeria"[All Fields]) OR rwandan[All Fields] OR (sao[All Fields] AND core[All Fields] AND principle[All Fields]) OR ("senegal"[MeSH Terms] OR "senegal"[All Fields]) OR ("seychelles"[MeSH Terms] OR "seychelles"[All Fields]) OR ("sierra leone"[MeSH Terms] OR ("sierra"[All Fields] AND "leone"[All Fields]) OR "sierra leone"[All Fields]) OR somali[All Fields] OR (british[All Fields] AND somaliland[All Fields]) OR ("south africa"[MeSH Terms] OR ("south"[All Fields] AND "africa"[All Fields]) OR "south africa"[All Fields]) OR ("south sudan"[MeSH Terms] OR ("south"[All Fields] AND "sudan"[All Fields]) OR "south sudan"[All Fields]) OR ("sudan"[MeSH Terms] OR "sudan"[All Fields]) OR (kingdom[All Fields] AND ("eswatini"[MeSH Terms] OR "eswatini"[All Fields] OR "swaziland"[All Fields])) OR ("eswatini"[MeSH Terms] OR "eswatini"[All Fields]) OR ("tanzania"[MeSH Terms] OR "tanzania"[All Fields]) OR ("togo"[MeSH Terms] OR "togo"[All Fields]) OR ("uganda"[MeSH Terms] OR "uganda"[All Fields]) OR ("zambia"[MeSH Terms] OR "zambia"[All Fields]) OR ("zimbabwe"[MeSH Terms] OR "zimbabwe"[All Fields]))) AND (("measurement"[All Fields] AND "month"[All Fields]) OR who-steps[All Fields] OR (("demography"[MeSH Terms] OR "demography"[All Fields] OR "demographic"[All Fields]) AND ("health surveys"[MeSH Terms] OR ("health"[All Fields] AND "surveys"[All Fields]) OR "health surveys"[All Fields] OR ("health"[All Fields] AND "survey"[All Fields]) OR "health survey"[All Fields])))) |
